# Supplementary material for: Inference of 3D genome architecture by modeling overdispersion of Hi-C data
Source: Bioinformatics. 2023 Jan 3;39(1):btac838. doi: 10.1093/bioinformatics/btac838 (PMC9857972; doi:10.1093/bioinformatics/btac838)
Supplement: btac838_Supplementary_Data [file btac838_supplementary_data.pdf]

Supplementaries materials

September 22, 2022

List of Tables

1 **3D inference methods** 9

2 **Percentage of loci filtered by dataset prior to normalization** 10

3 **Estimation of dispersion parameter w.r.t parameters.** Estimates of the dispersion parameter $\hat{r}$ for $l < \frac{2}{3} l_{\max}$ and $l < .99 l_{\max}$ 11

List of Figures

1 **Dispersion parameter as a function of genomic distance**. The dispersion parameter was estimated for each genomic distance. The black line corresponds to the dispersion parameter estimated for the entire dataset. 4

2 **Robustness to dispersion parameter.** The RMSD between groundtruth structure and inferred structures with miniMDS (the second best performing method on this dataset), and Pastis-NB with $\hat{r}/10$, $\hat{r}$, and $\hat{r} \times 10$. 5

3 **Stability of filtered versus unfiltered inference**. The Spearman correlation between distances matrices of pairs of structures inferred by Pastis-NB using all contact counts ("all") or excluding zero contact counts ("filtered"). 5

4 **Stability to coverage**. The Spearman correlation between distances matrices of a structure inferred at full resolution versus structures inferred on downsampled contact count matrices. . 5

5 **Multi-chromosomes structure inference.** The Spearman correlation between pairs of chromosomes inferred by subsampling chromosomes from Duan et al. [2010]'s *S. cerevisiae* dataset. 6

6 **Inferred models of chromosome 1 of KBM7.** Predicted models of KBM7's chromosome 1 (75 replicate Rao et al. [2014]) with ShNeigh (**A**, **C**) and Pastis-NB (**B**, **D**) at 250 kb (**A**, **B**) and 50 kb (**C**, **D**) 7

7 ***Inferred models of S. cerevisiae.*** Predicted models of *S. cerevisiae* with MDS (a), miniMDS (b), ShRec3D (c), ChromSDE (d), SuperRec (e), Pastis-PM (f), and Pastis-NB (g). Each color corresponds to a single chromosome, white beads to telomeres. 8

Contents

1 Supplementary materials **3**

1.1 Simulated datasets 3

1.2 Real datasets 3

1.3 Measures of 3D model similarities 3

2 Supplementary Figures **4**

3 Supplementary tables **9**

4 Supplementary materials **11**

4.1 Normalization of the data 11

1 Supplementary materials

1.1 Simulated datasets

Because little experimental data is available to characterize the true population of 3D DNA structure, we first compare the different structural inference methods by using simulated data. We construct three ensembles of datasets with varying coverage, dispersion, and counts-to-distance mapping. All simulations use a consensus architecture obtained by running Pastis-MDS, applied to the first chromosome of the 75th replicate of the KBM7 nearly haploid human cell line data from Rao *et al.* Rao et al. [2014] at 100 kb.

We generate simulated datasets using the model $C_{ij} \sim \text{NB}(\beta d_{ij}^{\alpha}, \beta r)$. Note that we do not simulate biased data requiring ICE normalization, to focus on the architecture inference part. Since d_{ij} is given by the consensus architecture used to simulate the counts, for all pairs of beads (i, j), the total number of counts in the count matrix (a.k.a. coverage) is on average $K = \beta \sum_{i,j} d_{ij}^{\alpha}$; hence we control the coverage in a simulation with the β parameter by setting $\beta = K / \sum_{i,j} d_{ij}^{\alpha}$.

We generate the first ensemble of 100 datasets to study the influence of coverage. We use $\alpha = -3$, which yields a count-to-distance mapping consistent with the one obtained from polymer physics theory [Grosberg et al., 1988, Lieberman-Aiden et al., 2009, Fudenberg and Mirny, 2012]. We vary the parameter β such that the expected number of reads ranges between 10% and 100% of the original dataset (10%, 20%, 30%, 40%, 50%, 60%, 70%, 80%, 90%, and 100%), and we set the dispersion parameter r to be the one fitted as described above to the KBM7 Hi-C dataset ($r = 49.9$), obtaining 100 datasets by repeating each configuration 10 times with 10 random seeds.

The second collection of 100 simulated datasets is to study the influence of overdispersion. We keep $\alpha = -3$ and set the parameter β such that the expected number of reads is 100% of the original dataset. We set the dispersion parameter r to be the one estimated on the original KBM7 contact maps multiplied by γ, where $\gamma \in \{0.1, 0.2, \dots, 1\}$. Varying γ thus varies the dispersion, and the smaller the dispersion parameter is, the more overdispersed the datasets are, and thus the harder the inference is likely to be. For each set of parameters, we generate 10 datasets using 10 different random seeds, thus yielding 100 datasets.

Finally, we generate an ensemble of 70 datasets to measure how well methods perform when provided with an incorrect counts-to-distances mapping. To do so, we vary $\alpha \in \{-1.5, -.2, \cdots -4., -4.5\}$, fix β for each simulation so that the number of reads is as the original dataset, keep the dispersion parameter r as fitted on the KBM7 Hi-C dataset, and repeat each simulation 10 times using 10 random seeds. This third ensemble of datasets enables us to compare *metric* methods, for which the counts-to-distance mapping is fixed or provided by the user *a priori*, versus *non-metric* methods, for which the counts-to-distance mapping is inferred jointly with the structure from the data.

1.2 Real datasets

We also apply our method to publicly available Hi-C data from the chronic myelogenous leukemia cell-line KBM7 [Rao et al., 2014], and whole-genome (already processed) Hi-C datasets from *S. cerevisiae* [Duan et al., 2010], *D. melanogaster* [Sexton et al., 2012], and *A. thaliana* [Feng et al., 2014].

The KBM7 cell line has the nice property of being nearly haploid: apart from chr 8 and a small part of chr 15, all chromosomes are haploid. We downloaded the first two replicates (experiment 75 and 76) and processed the data with HiC-Pro [Servant et al., 2015] to obtain intra-chromosomal maps at 1 Mb, 500 kb, 250 kb, 100 kb, and 50 kb.

1.3 Measures of 3D model similarities

To assess how well methods work, one needs to use similarity measures of 3D structures. In this work, we use two such measures: (1) the root mean square deviation (RMSD) and the Spearman correlation.

The RMSD is a commonly used metric to compare two structures $X \in \mathbb{R}^{3 \times n}$ and $X' \in \mathbb{R}^{3 \times n}$. It is defined as

$$RMSD(X, X') = \min_{s,R,t} \sqrt{\sum_{i=1}^{n} \left((x_i - (sRx'_i - t)\right)^2},$$

where $s \in \mathbb{R}$ is a scaling factor, $R \in \mathbb{R}^{3\times3}$ is a rotation matrix, and $t \in \mathbb{R}^3$ is a translation factor. The RMSD is sensitive to the scale of the structure X. Thus, when the scale of X is not known, we apply a robust rescaling strategy to X, such that 99% of the beads fit into a sphere of a predefined diameter. Note that the RMSD will be greatly affected by the presence of outlier beads. High RMSD indicates a poor reconstruction.

The second measure of similarity we use is the Spearman correlation of pairwise distances of the two structures. The Spearman correlation metric is much more robust to outliers than RMSD. Thus, some methods could have high RMSD in the presence of outliers but still achieve a high Spearman correlation.

2 Supplementary Figures

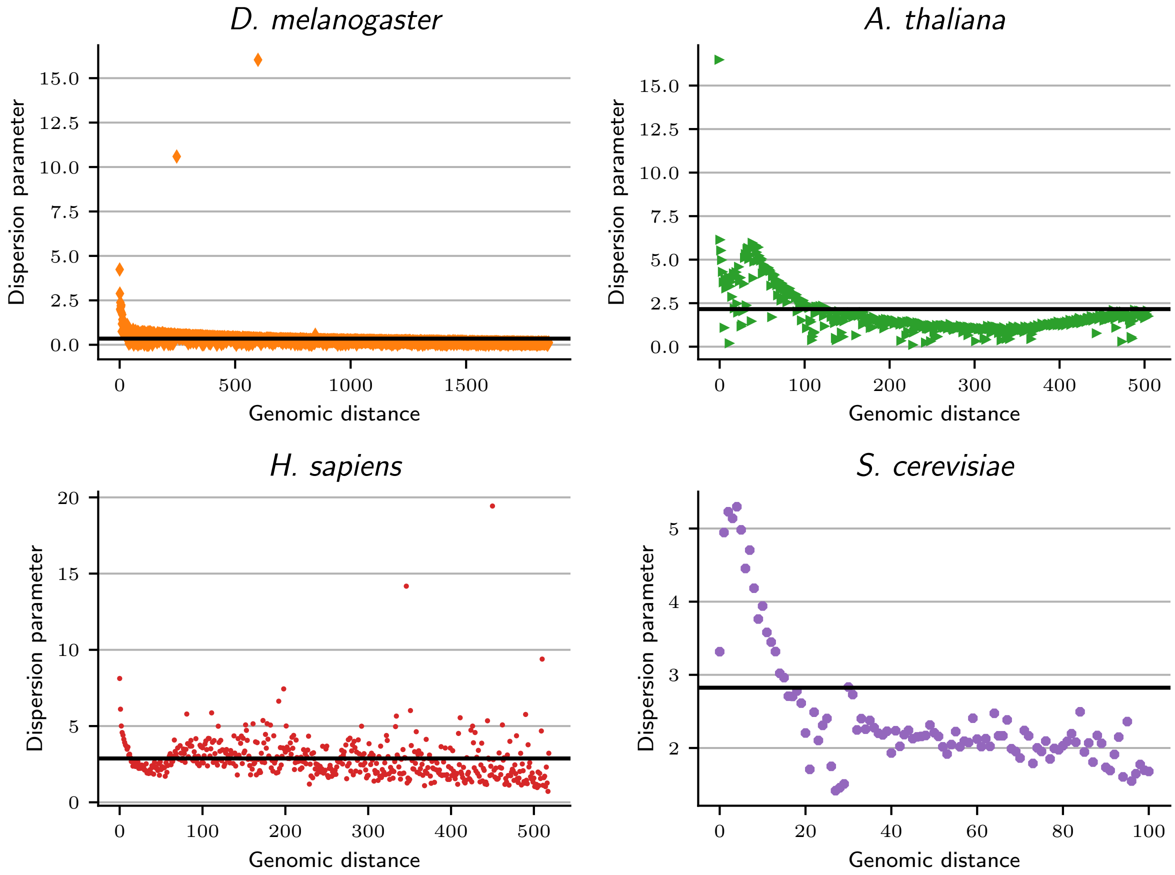

Supplementary Figure 1: Dispersion parameter as a function of genomic distance. The dispersion parameter was estimated for each genomic distance. The black line corresponds to the dispersion parameter estimated for the entire dataset.

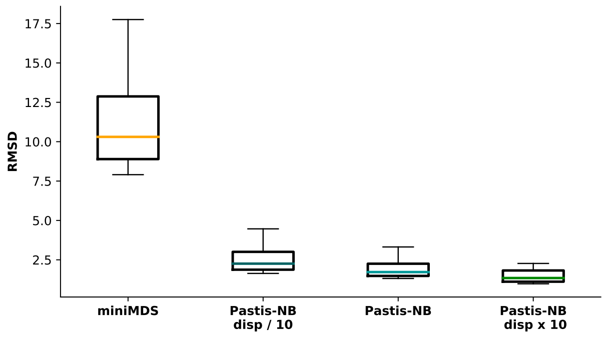

Supplementary Figure 2: Robustness to dispersion parameter. The RMSD between groundtruth structure and inferred structures with miniMDS (the second best performing method on this dataset), and Pastis-NB with $\hat{r}/10$, $\hat{r}$, and $\hat{r} \times 10$.

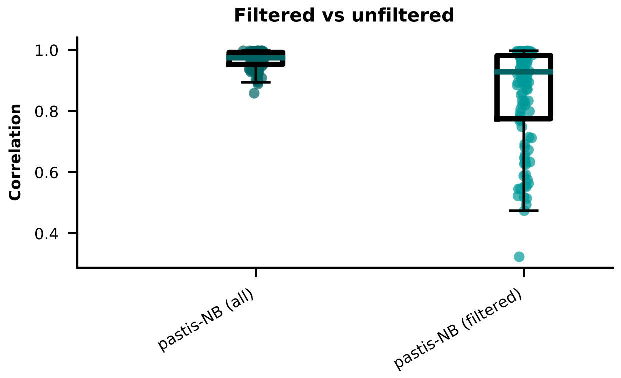

Supplementary Figure 3: Stability of filtered versus unfiltered inference. The Spearman correlation between distances matrices of pairs of structures inferred by Pastis-NB using all contact counts ("all") or excluding zero contact counts ("filtered").

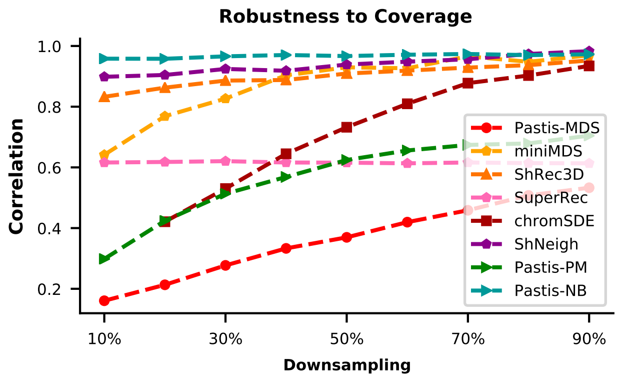

Supplementary Figure 4: Stability to coverage. The Spearman correlation between distances matrices of a structure inferred at full resolution versus structures inferred on downsampled contact count matrices.

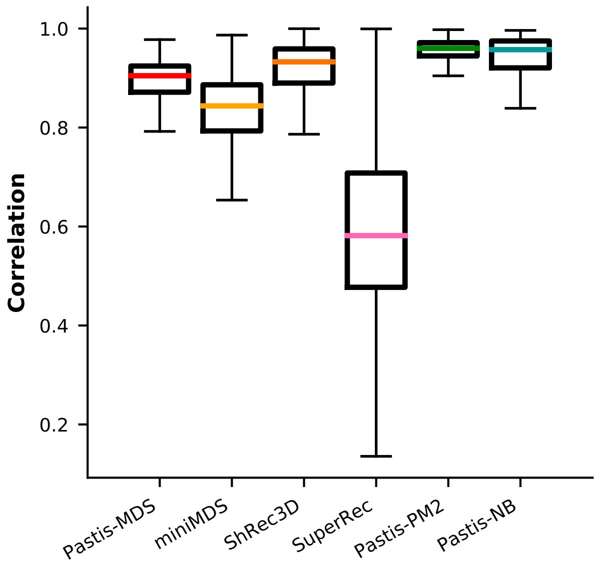

Supplementary Figure 5: Multi-chromosomes structure inference. The Spearman correlation between pairs of chromosomes inferred by subsampling chromosomes from Duan et al. [2010]'s *S. cerevisiae* dataset.

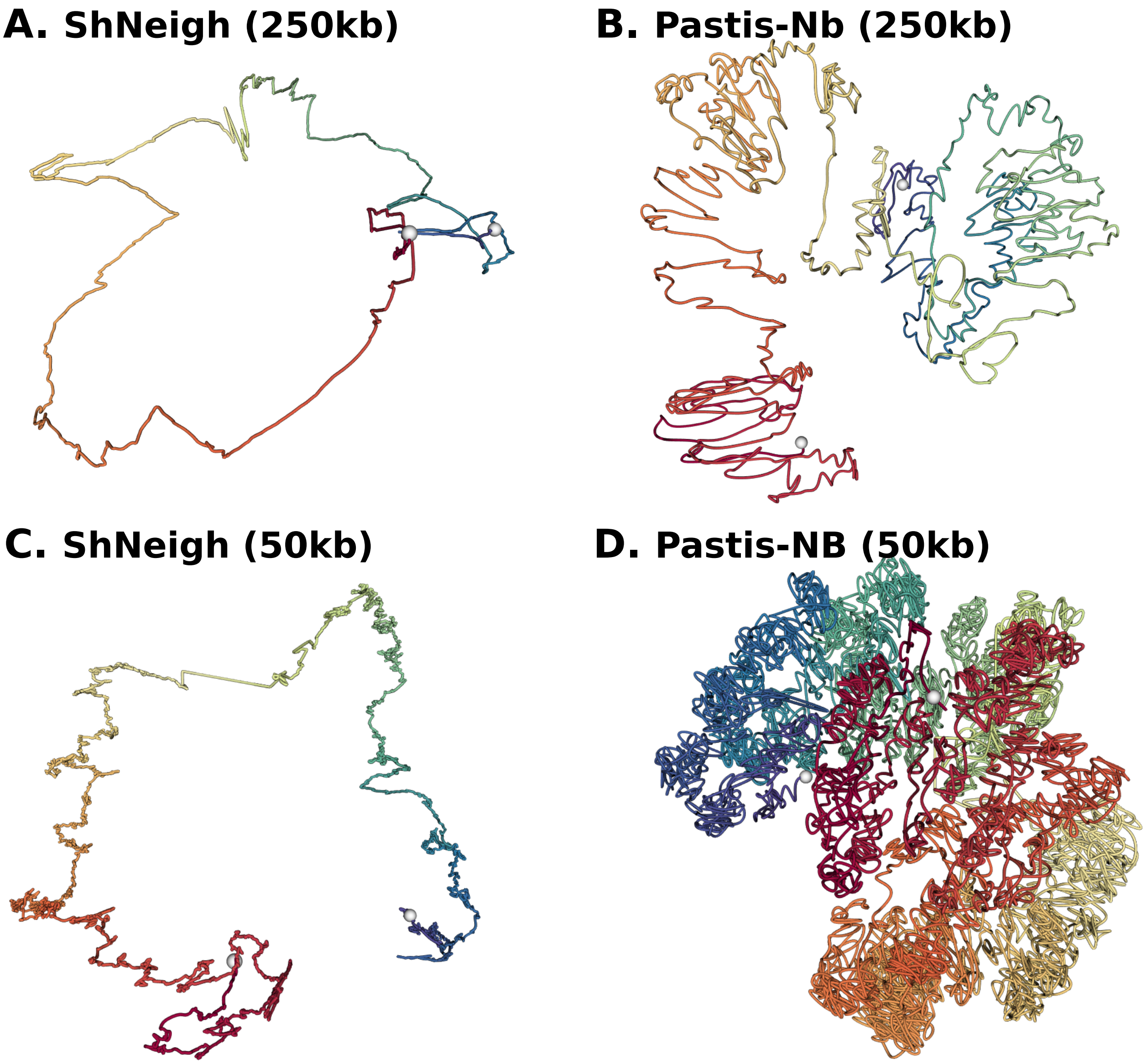

Supplementary Figure 6: Inferred models of chromosome 1 of KBM7. Predicted models of KBM7's chromosome 1 (75 replicate Rao et al. [2014]) with ShNeigh (A, C) and Pastis-NB (B, D) at 250 kb (A, B) and 50 kb (C, D)

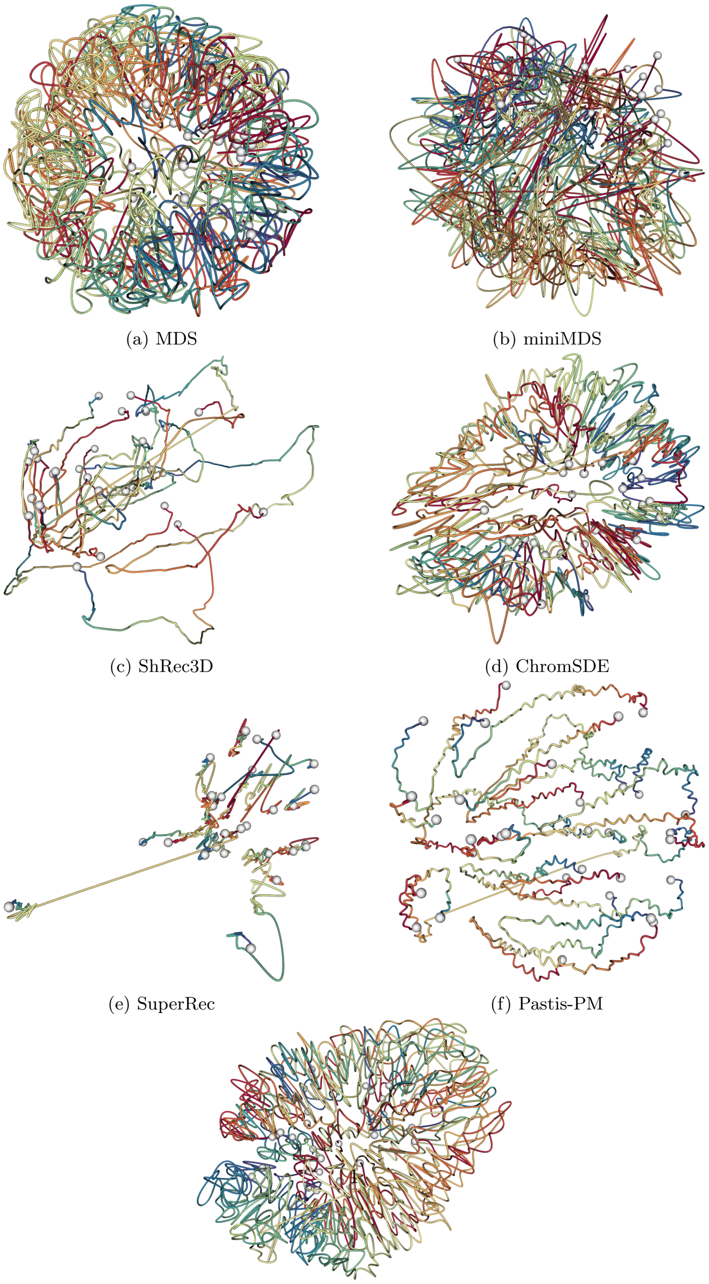

(a) MDS (b) miniMDS

(c) ShRec3D (d) ChromSDE

(e) SuperRec (f) Pastis-PM

(g) Pastis-NB

Supplementary Figure 7: Inferred models of *S. cerevisiae*. Predicted models of *S. cerevisiae* with MDS (a), miniMDS (b), ShRec3D (c), ChromSDE (d), SuperRec (e), Pastis-PM (f), and Pastis-NB (g). Each color corresponds to a single chromosome, white beads to telomeres.

3 Supplementary tables

Publication	Name	*Con.* or *Ens.*	Type	Available	Comment
Dekker et al. [2002]		C	MDS		Organism specific
Duan et al. [2010]		C	MDS	✓(C++)	Organism specific
Tanizawa et al. [2010]		C	MDS		
Baù et al. [2011]		E			
Rousseau et al. [2011]	MCMC5	E	Statistical	✓*	
Umbarger et al. [2011]		E			
Kalhor et al. [2011]		E			
**Zhang et al. [2013]**	chromSDE	C	MDS	✓(matlab)	
Peng et al. [2013]	autochrom3D	C	MDS	✓(Perl)	
Ben-Elazar et al. [2013]		C	MDS		Not available
Nagano et al. [2013]		E			
Hu et al. [2013]	Bach	E/C	Statistical	✓*	Not available
Ay et al. [2014]		C	MDS		Organism specific
**Varoquaux et al. [2014]**	Pastis	C	Statistical	✓(Python)	
**Lesne et al. [2014]**	ShRec3D	C	MDS	✓(matlab)	
Trieu and Cheng [2014]		C			
Diament and Tuller [2015]			MDS	(C++)	Not available
Wang et al. [2015]	InfMod3DGen	E	Bayesian	✓(matlab)	
Paulsen et al. [2015]	MBO	C	MDS (single-cell)	✓*	
Segal and Bengtsson [2015]		C			Not available
Caudai et al. [2015]		C		✓* (Python)	Not available
Zou et al. [2016]	HSA	C		✓(R)	for multi-track HiC
Adhikari et al. [2016]	Chromosome3D	C		✓* (Perl)	Not available
Trieu and Cheng [2016]	MOGEN	C			Not available
Carstens et al. [2016]	ISDHiC	C	(single-cell)	✓(Python)	
Hirata et al. [2016]	RPR	C	MDS (single-cell)		
Trieu and Cheng [2017]		C		✓*	Not available
**Rieber and Mahony [2017]**	minimds	C	MDS	✓(Python)	
Paulsen et al. [2017]	Chrom3D	C			HiC + lamine data
Li et al. [2018]	SchRec3D+	C	MDS		Not available
Zhu et al. [2018]	GEM	E	manifold learning	✓(matlab)	
**Zhang et al. [2019]**	SuperRec	C	MDS	✓*	Source not available
Kapilevich et al. [2019]	MDSGA	C	MDS	✓* (Python)	For simulation data only
Cauer et al. [2019]	Pastis	C	Statistical (diploid)	✓(Python)	
Trieu et al. [2018]	GenomeFlow	C			Graphical tool
Trieu et al. [2019]	Hierarchical3DGenome	C		✓(java)	Relies on TAD
**Li et al. [2020]**	Schneigh	C	MDS	✓(matlab)	
Ye and Ma [2020]	ASHIC	C	Statistical (diploid)	✓(Python)	

Supplementary Table 1: 3D inference methods

Incomplete list of methods to perform 3D structure inference from Hi-C data (1) is it a *consensus* or a *ensemble* based inference? (2) What type of methods is it (MDS-based, statistical modeling, or others)? (3) is the software available or not (to the best of our knowledge)? (4) What programming language is it written in? Software with availability marked with a checkmark and an asterisk indicates either a problem with the link provided in the article or partial availability (*i.e.*, SuperRec's code is not publicly available, but a binary can be downloaded and used on linux platforms). Rows highlighted in yellow corresponds to methods comparable in scope with Pastis-NB (infers non-diploid consensus structures from bulk HiC data and are none-organisms specific). In bold are the methods selected for comparison in this paper.

Dataset	% loci filtered
Rao et al. [2014] (1 mb)	1%
Rao et al. [2014] (500 kb)	2%
Rao et al. [2014] (250 kb)	3%
Rao et al. [2014] (200 kb)	4%
Rao et al. [2014] (100 kb)	5%
Rao et al. [2014] (50 kb)	6%
Duan et al. [2010] (10 kb)	4%
Sexton et al. [2012] (10 kb)	4%
Feng et al. [2014] (40 kb)	4%

Supplementary Table 2: Percentage of loci filtered by dataset prior to normalization

	D. melanogaster	*A. thaliana*	*H. sapiens*	*S. cerevisiae*
$\frac{2}{3}l_{\max}$	0.34	2.16	2.87	2.82
$.99l_{\max}$	0.33	2.19	2.67	2.80

Supplementary Table 3: Estimation of dispersion parameter w.r.t parameters. Estimates of the dispersion parameter $\hat{r}$ for $l < \frac{2}{3}l_{\max}$ and $l < .99l_{\max}$

4 Supplementary materials

4.1 Normalization of the data

The raw contact count matrix $\mathbf{c}$ suffers from many biases, some technical (from the sequencing and mapping) and others biological (inherent in the physical properties of chromatin) [Imakaev et al., 2012, Yaffe and Tanay, 2011]. Imakaev et al. [2012] proposed a simple iterative correction and eigenvalue decomposition method called ICE to estimate the biases and normalize the data. This method relies on two primary assumptions. First, the biases of each entry c_{ij} can be written as the product of two biases b_i and b_j associated with loci i and j; thus, if $\mathbf{c}^N$ is the normalized contact count matrix, then $c_{ij} = b_i b_j c_{ij}^N$. Second, the total number of contacts associated with each locus should be equal. From these assumptions, we can formulate a non-convex optimization problem to estimate the vector of biases b. The problem can be solved exactly using an iterative procedure akin to the Sinkhorn algorithm. We apply the ICE method to all of the data used in this study. Prior to normalization, we filter out rows and columns having the fewest number of contacts to avoid degeneracies during the iterative correction (see Supplementary Table 2).

References

B. Adhikari, T. Trieu, and J. Cheng. Chromosome3D: reconstructing three-dimensional chromosomal structures from Hi-C interaction frequency data using distance geometry simulated annealing. *BMC Genomics*, 17(1):886, 11 2016.

F. Ay, E. M. Bunnik, N. Varoquaux, S. M. Bol, J. Prudhomme, J.-P. Vert, W. S. Noble, and K. G. Le Roch. Three-dimensional modeling of the *P. falciparum* genome during the erythrocytic cycle reveals a strong connection between genome architecture and gene expression. *Genome Research*, 24:974–988, 2014.

D. Baù, A. Sanyal, B. R. Lajoie, E. Capriotti, M. Byron, J. B. Lawrence, J. Dekker, and M. A. Marti-Renom. The three-dimensional folding of the α-globin gene domain reveals formation of chromatin globules. *Nat Struct Mol Biol*, 18(1):107–114, 2011.

S. Ben-Elazar, Z. Yakhini, and I. Yanai. Spatial localization of co-regulated genes exceeds genomic gene clustering in the saccharomyces cerevisiae genome. *Nucleic Acids Res*, 41(4):2191–2201, Feb 2013.

S. Carstens, M. Nilges, and M. Habeck. Inferential Structure Determination of Chromosomes from Single-Cell Hi-C Data. *PLoS Comput Biol*, 12(12):e1005292, Dec 2016.

C. Caudai, E. Salerno, M. Zoppè, and A. Tonazzini. Inferring 3D chromatin structure using a multiscale approach based on quaternions. *BMC Bioinformatics*, 16:234, Jul 2015.

A. G. Cauer, G. Yardimci, J.-P. Vert, N. Varoquaux, and W. S. Noble. Inferring Diploid 3D Chromatin Structures from Hi-C Data. In K. T. Huber and D. Gusfield, editors, *19th International Workshop on Algorithms in Bioinformatics (WABI 2019)*, volume 143 of *Leibniz International Proceedings in Informatics (LIPIcs)*, pages 11:1–11:13, Dagstuhl, Germany, 2019. Schloss Dagstuhl–Leibniz-Zentrum fuer Informatik. ISBN 978-3-95977-123-8. doi: 10.4230/LIPIcs.WABI.2019.11.

J. Dekker, K. Rippe, M. Dekker, and N. Kleckner. Capturing chromosome conformation. *Science*, 295(5558):1306–1311, 2002.

A. Diament and T. Tuller. Improving 3d genome reconstructions using orthologous and functional constraints. *PLOS Computational Biology*, 11(5):1–22, 05 2015. doi: 10.1371/journal.pcbi.1004298. URL `https://doi.org/10.1371/journal.pcbi.1004298`.

Z. Duan, M. Andronescu, K. Schutz, S. McIlwain, Y. J. Kim, C. Lee, J. Shendure, S. Fields, C. A. Blau, and W. S. Noble. A three-dimensional model of the yeast genome. *Nature*, 465:363–367, 2010.

S. Feng, S. J. Cokus, V. Schubert, J. Zhai, M. Pellegrini, and S. E. Jacobsen. Genome-wide Hi-C analyses in wild-type and mutants reveal high-resolution chromatin interactions in *Arabidopsis*. *Mol. Cell*, 55(5):694–707, Sep 2014.

G. Fudenberg and L. A. Mirny. Higher-order chromatin structure: bridging physics and biology. *Curr Opin Genet Dev.*, 22(2):115–124, 2012.

A. Y. Grosberg, S. K. Nechaev, and E. I. Shakhnovich. The role of topological constraints in the kinetics of collapse of macromolecules. *Journal de Physique*, 49(12):2095–2100, 1988.

Y. Hirata, A. Oda, K. Ohta, and K. Aihara. Three-dimensional reconstruction of single-cell chromosome structure using recurrence plots. *Sci Rep*, 6:34982, Oct 2016.

M. Hu, K. Deng, Z. Qin, J. Dixon, S. Selvaraj, J. Fang, B. Ren, and J. S. Liu. Bayesian inference of spatial organizations of chromosomes. *PLoS Comput Biol*, 9(1):e1002893, 2013.

M. Imakaev, G. Fudenberg, R. P. McCord, N. Naumova, A. Goloborodko, B. R. Lajoie, J. Dekker, and L. A. Mirny. Iterative correction of Hi-C data reveals hallmarks of chromosome organization. *Nature Methods*, 9:999–1003, 2012.

R. Kalhor, H. Tjong, N. Jayathilaka, F. Alber, and L. Chen. Genome architectures revealed by tethered chromosome conformation capture and population-based modeling. *Nat Biotechnol*, 2011.

V. Kapilevich, S. Seno, H. Matsuda, and Y. Takenaka. Chromatin 3D Reconstruction from Chromosomal Contacts Using a Genetic Algorithm. *IEEE/ACM Trans Comput Biol Bioinform*, 16(5):1620–1626, 2019.

A. Lesne, J. Riposo, A. Roger, Pauland Cournac, and J. Mozziconacci. 3D genome reconstruction from chromosomal contacts. *Nature Methods*, 11(11):1141–1143, 2014.

F.-Z. Li, Z.-E. Liu, X.-Y. Li, L.-M. Bu, H.-X. Bu, H. Liu, and C.-M. Zhang. Chromatin 3D structure reconstruction with consideration of adjacency relationship among genomic loci. *BMC Bioinformatics*, 21(1):272, Jul 2020.

J. Li, W. Zhang, and X. Li. 3D Genome Reconstruction with ShRec3D+ and Hi-C Data. *IEEE/ACM Trans Comput Biol Bioinform*, 15(2):460–468, 2018.

E. Lieberman-Aiden, N. L. van Berkum, L. Williams, M. Imakaev, T. Ragoczy, A. Telling, I. Amit, B. R. Lajoie, P. J. Sabo, M. O. Dorschner, R. Sandstrom, B. Bernstein, M. A. Bender, M. Groudine, A. Gnirke, J. Stamatoyannopoulos, L. A. Mirny, E. S. Lander, and J. Dekker. Comprehensive mapping of long-range interactions reveals folding principles of the human genome. *Science*, 2009.

T. Nagano, Y. Lubling, T. J. Stevens, S. Schoenfelder, E. Yaffe, W. Dean, E. D. Laue, A. Tanay, and P. Fraser. Single-cell Hi-C reveals cell-to-cell variability in chromosome structure. *Nature*, 2013.

J. Paulsen, O. Gramstad, and P. Collas. Manifold based optimization for single-cell 3d genome reconstruction. *PLoS Comput Biol*, 11 (8):e1004396, 08 2015. doi: 10.1371/journal.pcbi.1004396. URL http://dx.doi.org/10.1371%2Fjournal.pcbi.1004396.

J. Paulsen, M. Sekelja, A. R. Oldenburg, A. Barateau, N. Briand, E. Delbarre, A. Shah, A. L. Sørensen, C. Vigouroux, B. Buendia, and P. Collas. Chrom3D: three-dimensional genome modeling from Hi-C and nuclear lamin-genome contacts. *Genome Biol*, 18(1): 21, 01 2017.

C. Peng, L.-Y. Fu, P.-F. Dong, Z.-L. Deng, J.-X. Li, X.-T. Wang, and H.-Y. Zhang. The sequencing bias relaxed characteristics of Hi-C derived data and implications for chromatin 3D modeling. *Nucleic Acids Res.*, 41(19):e183, 2013.

S. P. Rao, M. H. Huntley, N. Durand, E. K. Stamenova, I. D. Bochkov, J. T. Robinson, A. L. Sanborn, I. Machol, A. D. Omer, E. S. Lander, and E. Lieberman-Aiden. A 3D map of the human genome at kilobase resolution reveals principles of chromatin looping. *Cell*, 2014.

L. Rieber and S. Mahony. miniMDS: 3D structural inference from high-resolution Hi-C data. *Bioinformatics*, 33(14):i261–i266, Jul 2017.

M. Rousseau, J. Fraser, M. Ferraiuolo, J. Dostie, and M. Blanchette. Three-dimensional modeling of chromatin structure from interaction frequency data using Markov chain Monte Carlo sampling. *BMC Bioinformatics*, 12(1):414, Oct. 2011. ISSN 1471-2105.

M. R. Segal and H. L. Bengtsson. Reconstruction of 3D genome architecture via a two-stage algorithm. *BMC Bioinformatics*, 16:373, Nov 2015.

N. Servant, N. Varoquaux, B. R. Lajoie, E. Viara, C.-J. Chen, J.-P. Vert, E. Heard, J. Dekker, and E. Barillot. HiC-Pro: an optimized and flexible pipeline for Hi-C data processing. *Genome Biol.*, 16:259, 2015.

T. Sexton, E. Yaffe, E. Kenigsberg, F. Bantignies, B. Leblanc, M. Hoichman, H. Parrinello, A. Tanay, and G. Cavalli. Three-dimensional folding and functional organization principles of the *Drosophila* genome. *Cell*, 2012.

H. Tanizawa, O. Iwasaki, A. Tanaka, J. R. Capizzi, P. Wickramasignhe, M. Lee, Z. Fu, and K.-i. Noma. Mapping of long-range associations throughout the fission yeast genome reveals global genome organization linked to transcriptional regulation. *Nucleic Acids Res*, 38(22):8164–8177, 2010.

T. Trieu and J. Cheng. Large-scale reconstruction of 3D structures of human chromosomes from chromosomal contact data. *Nucleic Acids Res.*, 42(7):e52, 2014.

T. Trieu and J. Cheng. MOGEN: a tool for reconstructing 3D models of genomes from chromosomal conformation capturing data. *Bioinformatics*, 32(9):1286–1292, May 2016.

T. Trieu and J. Cheng. 3D genome structure modeling by Lorentzian objective function. *Nucleic Acids Res.*, 45(3):1049–1058, 2017.

T. Trieu, O. Oluwadare, J. Wopata, and J. Cheng. GenomeFlow: a comprehensive graphical tool for modeling and analyzing 3D genome structure. *Bioinformatics*, 35(8):1416–1418, 09 2018. ISSN 1367-4803. doi: 10.1093/bioinformatics/bty802. URL `https://doi.org/10.1093/bioinformatics/bty802`.

T. Trieu, O. Oluwadare, and J. Cheng. Hierarchical Reconstruction of High-Resolution 3D Models of Large Chromosomes. *Sci Rep*, 9 (1):4971, 03 2019.

M. A. Umbarger, E. Toro, M. A. Wright, G. J. Porreca, D. Baù, S.-H. Hong, M. J. Fero, L. J. Zhu, M. A. Marti-Renom, H. H. McAdams, L. Shapiro, J. Dekker, and G. M. Church. The three-dimensional architecture of a bacterial genome and its alteration by genetic perturbation. *Molecular Cell*, 44:252–264, 2011.

N. Varoquaux, F. Ay, W. S. Noble, and J.-P. Vert. A statistical approach for inferring the 3D structure of the genome. *Bioinformatics*, 2014.

S. Wang, J. Xu, and J. Zeng. Inferential modeling of 3D chromatin structure. *Nucleic Acids Research*, 43(8):e54–e54, 02 2015. ISSN 0305-1048. doi: 10.1093/nar/gkv100. URL `https://doi.org/10.1093/nar/gkv100`.

E. Yaffe and A. Tanay. Probabilistic modeling of Hi-C contact maps eliminates systematic biases to characterize global chromosomal architecture. *Nat Genet*, 43:1059–1065, 2011.

T. Ye and W. Ma. ASHIC: hierarchical Bayesian modeling of diploid chromatin contacts and structures. *Nucleic Acids Res*, 48(21): e123, 12 2020.

Y. Zhang, W. Liu, Y. Lin, Y. K. Ng, and S. Li. Large-scale 3D chromatin reconstruction from chromosomal contacts. *BMC Genomics*, 20(Suppl 2):186, Apr 2019.

Z. Zhang, G. Li, K.-C. Toh, and W.-K. Sung. Inference of spatial organizations of chromosomes using semi-definite embedding approach and Hi-C data. In *Proceedings of the 17th International Conference on Research in Computational Molecular Biology*, volume 7821 of *Lecture Notes in Computer Science*, pages 317–332, Berlin, Heidelberg, 2013. Springer-Verlag.

G. Zhu, W. Deng, H. Hu, R. Ma, S. Zhang, J. Yang, J. Peng, T. Kaplan, and J. Zeng. Reconstructing spatial organizations of chromosomes through manifold learning. *Nucleic Acids Res*, 46(8):e50, 05 2018.

C. Zou, Y. Zhang, and Z. Ouyang. HSA: integrating multi-track Hi-C data for genome-scale reconstruction of 3D chromatin structure. *Genome Biol*, 17:40, Mar 2016.
